# Corporate Ownership, Health System Affiliation, and Market Concentration of Home Health Agencies

**DOI:** 10.1001/jamanetworkopen.2025.28258

**Published:** 2025-08-21

**Authors:** Kun Li, Shekinah Fashaw-Walters, M. Kate Bundorf, Yucheng Hou

**Affiliations:** 1 Duke-Margolis Institute for Health Policy, Washington, DC and Durham, North Carolina; 2 Department of Medical Ethics and Health Policy, Perelman School of Medicine, University of Pennsylvania, Philadelphia; 3 Sanford School of Public Policy, Duke University, Durham, North Carolina; 4 Department of Management, Policy and Community Health, School of Public Health, The University of Texas Health Science Center at Houston, Houston

## Abstract

This cross-sectional study examines how growing health system affiliation and corporate ownership models may potentially contribute to home health agency (HHA) market concentration.

## Introduction

The home health agency (HHA) industry has been growing substantially, with spending growth on home health services outpacing that of nursing homes since 2000.^1,2^ During this time, health systems and corporate investors have increasingly acquired HHAs.^2,3^ While acquisitions may facilitate care coordination, they may also lead to fewer owners controlling the market, raising concerns over the quality of and spending on home health services. We provide new data on how growing system affiliation and corporate ownership may contribute to HHA market concentration.

## Methods

This cross-sectional study, which follows the STROBE reporting guideline, measured HHA market concentration in 2022 using the Herfindahl-Hirschman Index (HHI). We defined HHA markets using the county-based definition of health service areas (hereafter, *markets*) developed by the National Center for Health Statistics ( eMethods in  Supplement 1).^4^ Market shares were calculated on the basis of HHAs’ Medicare fee-for-service standardized payment amount reported in the Medicare Post-Acute Care & Hospice Public Use Files; we tested alternative measures for market share: number of fee-for-service Medicare patients and total service days.

We calculated 3 types of HHIs: agency-level (unadjusted), system-adjusted, and owner-adjusted. Using the 2022 Compendium of US Health System Home Health Care Organization linkage file, we associated HHAs with health systems and corporate owners (including health systems) (eMethods in Supplement 1). We calculated system-adjusted and owner-adjusted HHIs by grouping HHAs affiliated with the same health system or corporate owner within a market as a single entity when calculating market shares. We described distributions of HHIs and the number of HHA owners that controlled 50%, 75%, and 100% market shares, separately for metropolitan and nonmetropolitan markets. Data were analyzed using Stata, version 18.0. Institutional review board approval and informed consent were not needed because no individual participant data were used per 45 CFR §46.

## Results

Our analytic sample included 5884 Medicare-certified HHAs in 2022: 3823 (65%) in 256 metropolitan markets and 2061 (35%) in 518 nonmetropolitan markets; 694 (12%) were affiliated with health systems and 2216 (38%) had corporate owners. Median (IQR) unadjusted HHIs were 2964 (1401-5059) in metropolitan markets and 5517 (3360-10 000) in nonmetropolitan markets (Table). Median (IQR) owner-adjusted HHIs were 3068 (1684-5236) and 5637 (3491-10 000) in metropolitan and nonmetropolitan markets, respectively. A total of 182 (71%) of metropolitan and 482 (93%) of nonmetropolitan markets were highly concentrated with owner-adjusted HHIs higher than 1800, the threshold identified in merger guidelines.^5^ The Figure shows that 60 (23%) of metropolitan and 291 (56%) of nonmetropolitan markets had 1 or 2 owners accounting for the entire HHA market; 179 (70%) of metropolitan and 483 (93%) of nonmetropolitan markets had 2 or fewer owners accounting for one-half of the market.

**Table.** Home Health Agency Market Concentration by Metropolitan and Nonmetropolitan Health Service Areas, 2022^**a**^

Measures of market share	Standardized payments		Medicare patients		Total service days	
	Nonmetropolitan (n = 518)	Metropolitan (n = 256)	Nonmetropolitan (n = 518)	Metropolitan (n = 256)	Nonmetropolitan (n = 518)	Metropolitan (n = 256)
**Agency-level unadjusted HHIs**						
** HHI of HHA markets, median (IQR)**	5517 (3360-10 000)	2964 (1401-5059)	5576 (3333-10 000)	2916 (1459-5006)	5602 (3361-10 000)	2987 (1427-5069)
Share of markets with HHIs higher than 1800, %	91	68	92	68	91	66
System-adjusted HHIs						
HHI of HHA markets, median (IQR)	5523 (3378-10 000)	3015 (1542-5059)	5587 (3333-10 000)	2967 (1508-5006)	5606 (3377-10 000)	3044 (1537-5069)
Share of markets with HHIs higher than 1800, %	92	68	92	70	92	67
Owner-adjusted HHIs						
HHI of HHA markets, median (IQR)	5637 (3491-10 000)	3068 (1684-5236)	5693 (3401-10 000)	3044 (1685-5114)	5672 (3479-10 000)	3055 (1673-5266)
Share of markets with HHIs higher than 1800, %	93	71	93	72	93	70

Abbreviations: HHA, home health agency; HHI, Herfindahl-Hirschman Index.

^a^ HHIs for 256 metropolitan Health Service Areas and 518 nonmetropolitan Health Service Areas were based on 5884 Medicare-certified home health agencies in 2022. System-adjusted HHIs account for the extent to which multiple HHAs are owned by a single health system; owner-adjusted HHIs account for both vertical integration between HHAs and health systems or horizontal integration between HHAs owned by the same organization.

**Figure.**
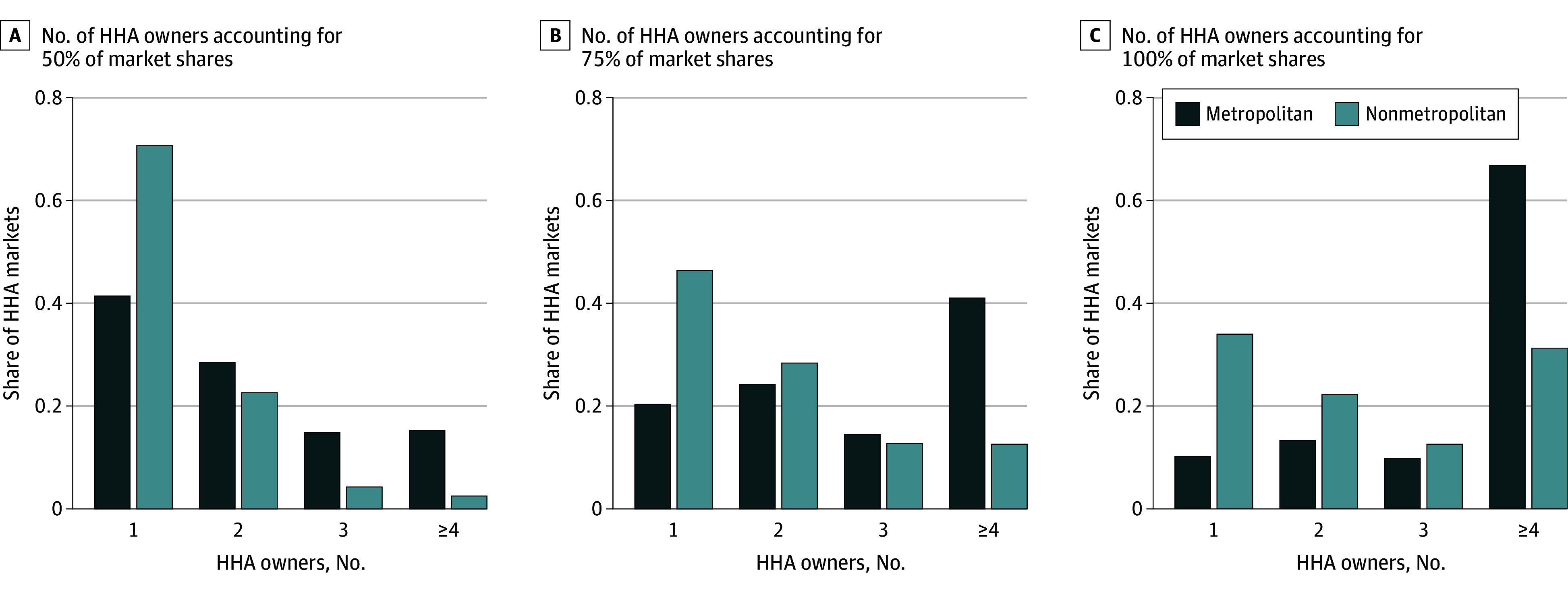
Share of Markets by Number of Home Health Agency (HHA) Owners, 2022 Graphs show the number of HHA owners controlling 50% (A), 75% (B), and 100% (C) of market shares in 256 metropolitan Health Service Areas and 518 nonmetropolitan health service areas on the basis of 5884 Medicare-certified home health agencies in 2022. Owner refers to an HHA corporate owner or an independent HHA, as applicable; the median number of owners was 5 for metropolitan markets and 2 for nonmetropolitan markets. Market shares were calculated as the share of Medicare fee-for-service standardized payment amount.

## Discussion

This cross-sectional study provides new data on the extent and structure of concentration in HHA markets. Even without adjusting for health systems or corporate owners, more than 80% of HHA markets were highly concentrated in 2022, with higher concentration among nonmetropolitan markets compared with metropolitan markets. Accounting for corporate ownership overall generated a larger increase in median HHIs than accounting for health system affiliation. Additionally, while more than one-half of the markets were served by 3 or more HHA owners, most markets had only 1 or 2 owners controlling a substantial proportion of the market share. These findings point to the importance of gaining a better understanding of the effects of concentration in the HHA market on health care spending, patient outcomes, and employment among home health workers.^6^ This evidence is essential for understanding the implications of and the role of policy in addressing increasing industry consolidation.

Limitations include that the data only identify Medicare-certified HHAs in the linked files and only capture their fee-for-service market shares. In addition, health service areas may not accurately capture home health worker travel patterns. Future studies using Medicare Advantage data and patient origin–based market measures to better understand the structure of markets for home health services are needed.

## Notes

Supplement 1.
eMethods.

eReferences

Supplement 2.
Data Sharing Statement
