# Supplementary material for: Corporate Ownership, Health System Affiliation, and Market Concentration of Home Health Agencies
Source: JAMA Netw Open. 2025 Aug 21;8(8):e2528258. doi: 10.1001/jamanetworkopen.2025.28258 (PMC12371508; doi:10.1001/jamanetworkopen.2025.28258)
Supplement: Supplement 1. — eMethods. eReferences [file jamanetwopen-e2528258-s001.pdf]

Supplemental Online Content

Li K, Fashaw-Walters S, Bundorf MK, Hou Y. Corporate ownership, health system affiliation, and market concentration of home health agencies. *JAMA Netw Open*. 2025;8(8):e2528258. doi:10.1001/jamanetworkopen.2025.28258

eMethods
eReferences

This supplemental material has been provided by the authors to give readers additional information about their work.

© 2025 Li K et al. *JAMA Network Open*.

Data Sources

AHRQ 2022 Compendium of U.S. Health System Home Health Care Organization Linkage File

The Agency for Healthcare Research and Quality (AHRQ) has created a linkage file to associate health systems or corporate parents with their member home healthcare organizations.[1] Home health care organizations included in the IQVIA OneKey data are broadly defined as organizations licensed to treat individuals in their home, including home health agencies (HHA) that provide skilled nursing and therapeutic services and other organizations specializing in home dialysis and/or hospice care.

According to the Compendium documentation, health systems include at least one hospital (non-federal acute care) and one group of primary care and specialty physicians (with at least 50 physicians and 10 primary care physicians) who relate to each other and the hospital through common ownership or joint management.[1] Health systems are categorized as one type of corporate parent in the data. Additionally, corporate parents also capture other organizational relationships (ownership, management, leasing, purchasing, and contracting mechanisms) reported in the OneKey data but did not meet the definition of health systems.[1]

Medicare Post-Acute Care and Hospice Public Use Files (PAC PUF)

The analysis is limited to HHAs in the OneKey data with matches in the 2022 Medicare Post-Acute Care and Hospice for HHAs, linked by the CMS Certification Number (CCN). The 2022 PAC PUF contains 8,547 Medicare-certified HHAs that submitted at least one Medicare Part A & B claim during the year,[2] of which we linked 5,884 (69%) HHAs in the Compendium. While system and corporate owner information is only available for the linked HHAs, we benchmarked our unadjusted HHIs using all Medicare-certified HHAs in the 2022 PAC PUF. The unadjusted HHIs are relatively stable.

Sensitivity Analysis	Herfindahl-Hirschman Index (HHI) of HHA Markets, Median<br>[25th percentile – 75th percentile]<br>Share of markets with HHIs higher than 1800, %					
Measures of Market Share	**Standardized payments**		**Medicare patients**		**Total service days**	
Agency-level Unadjusted HHIs	Nonmetropolitan (n=568)	Metropolitan (n=259)	Nonmetropolitan (n=568)	Metropolitan (n=259)	Nonmetropolitan (n=568)	Metropolitan (n=259)
	5,403 [3157-10000]	2,725 [1239-4680]	5,410 [3053-10000]	2,682 [1246-4670]	5,352 [3134-10000]	2,633 [1214-4702]
	90	64	90	64	90	64

Abbreviations: HHA, home health agency, HHI, Herfindahl-Hirschman Index.
Agency-level unadjusted HHIs for 259 metropolitan health service areas and 568 nonmetropolitan health services areas were based on 8506 Medicare-certified home health agencies in the 2022 Medicare Post-Acute Care and Hospice Public Use Files. Home health agencies in Puerto Rico and those with a zip code not mapping to a county (n=7) were excluded.

© 2025 Li K et al. *JAMA Network Open*.

Defining Home Health Market

To our knowledge, there has been very limited work addressing the definition of home health markets. There are several important features of home health care that need to be accounted for when defining the home health market. First, in a home health setting, providers travel to patients and deliver care in patients' homes. The market boundary of HHAs, therefore, should reflect the travel pattern of an average worker instead of that of patients for other types of medical care. Second, HHA staff are often not traveling from HHA's office but may be coming from different locations closer to the patient's home. As a result, patients who are not in metropolitan areas may be served by HHAs with an office located in metropolitan areas. Third, the low fixed costs of market entry and exit of HHAs have resulted in a high number of agencies clustered in some large metropolitan markets. These agencies may not directly compete with each other despite being located in the same metropolitan market.[3,4]

Given these considerations, we defined the geographic boundary of a home health market using the county-based definition of Health Service Areas developed by the National Center for Health Statistics.[5] The total of 952 Health Service Areas is comprised of relatively self-contained single counties or clusters of contiguous counties that capture patient commuting patterns for hospital care.[5] In the graph below, we delineated the Health Service Area boundary on top of the county lines. The travel distance within each market is close to the previously reported average travel distance between the HHA office and the patient's residence (with exceptions).[6]

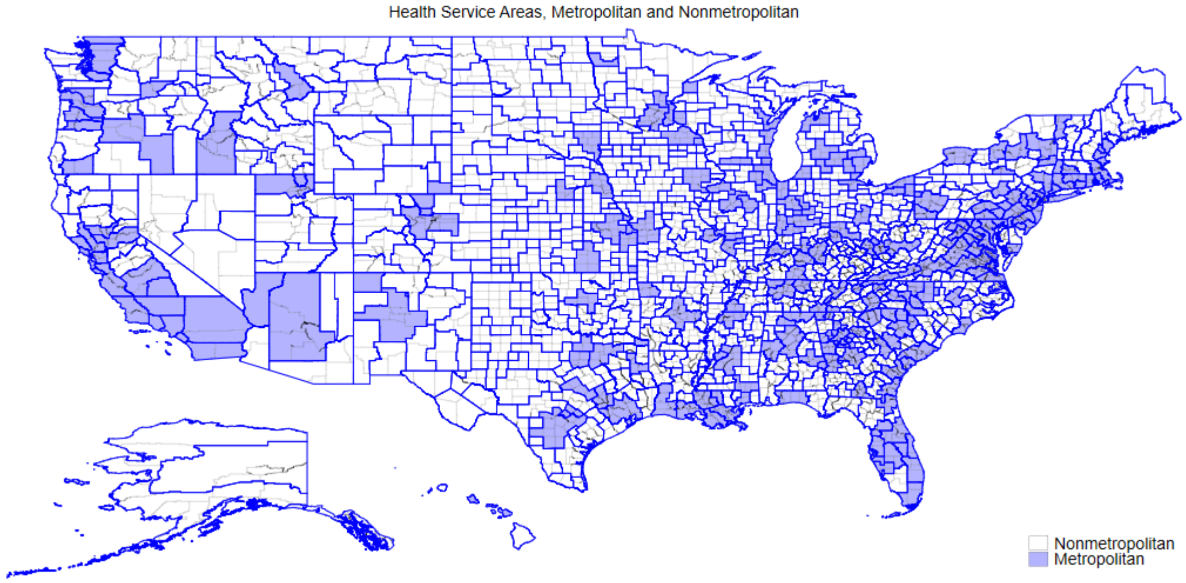

To assign HHA's ZIP codes to Health Service Areas (hereafter markets), we used the HUD-USPS ZIP code crosswalk files to assign ZIP codes to counties[7] and then counties to markets. When a ZIP code crossed county borders, we assigned that ZIP code to a primary county based on the highest proportion of all types of addresses that fall into each county in the overlapping area. We used the National Cancer Institute's modified crosswalk to assign counties to markets such that all counties from one market were in the same state.[5] We defined

© 2025 Li K et al. *JAMA Network Open*.

metropolitan and nonmetropolitan markets based on the county-level designation of Rural-Urban Continuum Codes (1-3, metropolitan counties; 4-9, nonmetropolitan counties).[8] Markets with at least 50% nonmetropolitan counties are designated as nonmetro markets, and metropolitan markets, vice versa. The number of metropolitan and nonmetropolitan markets does not sum to the total number of Health Service Areas because data only identify Medicare-certified HHAs in the linked files, and the HHA office may not be located in some markets.

	Total (n=5,884)	Nonmetro (n=2,061)	Metro (n=3,823)
No. of HHAs with a system affiliation (%)	694 (12)	305 (15)	389 (10)
No. of HHAs with a corporate owner (%)	2216 (38)	981 (48)	1235 (32)

Home Health Market Concentration Measure

We constructed the Herfindahl-Hirschman Index (HHI) to measure HHA market concentration, which is calculated as the sum of the squared market shares across all HHAs located in a home health market.[9] HHIs range from 0 to 10,000, with an HHI of zero representing a "perfectly competitive" market of infinite small HHAs and 10,000 representing a monopoly market of only one HHA controlling the entire market. We calculated system- and owner-adjusted HHIs by grouping HHAs affiliated with the same health system or corporate owner *within* a market as a single entity when calculating market shares. Since large systems and corporate owners can span market boundaries, we restructure the data so that each unit of analysis is a unique system-market or owner-market combination. HHA market share was calculated based on HHAs' Medicare fee-for-service standardized payment amounts; we also tested using the number of Medicare patients served and total service days to measure HHA market shares. Denoting *S* the set of HHAs, health systems, or HHA owners (when applicable), HHIs for metropolitan or nonmetropolitan markets *m* are given by:

$$HHI_m = \sum_{s=1}^{S} \left(\frac{payment_{s,m}}{\sum_S payment_{s,m}} \times 100 \right)^2$$

© 2025 Li K et al. *JAMA Network Open*.

eReferences

1. Agency for Healthcare Research and Quality. *Compendium of U.S. Health Systems, 2022, Home Health Care Organization Linkage File, Technical Documentation*.; 2024. Accessed November 14, 2024. https://www.ahrq.gov/sites/default/files/wysiwyg/chsp/compendium/2022-hhco-linkage-techdoc.pdf

2. Centers for Medicare & Medicaid Services. Medicare Post-Acute Care and Hospice Public Use Files. 2022. Accessed November 15, 2024. https://data.cms.gov/provider-summary-by-type-of-service/medicare-post-acute-care-hospice

3. Grabowski DC. The Market for Long-Term Care Services. *Inq J Health Care Organ Provis Financ*. 2008;45(1):58-74. doi:10.5034/inquiryjrnl_45.01.58

4. Jung K, Polsky D. Competition and Quality in Home Health Care Markets. *Health Econ*. 2014;23(3):298-313. doi:10.1002/hec.2938

5. National Cancer Institute SEER Program. Health Service Areas (HSA). April 19, 2023. Accessed June 17, 2025. https://seer.cancer.gov/seerstat/variables/countyattribs/hsa.html

6. Wang Y, Leifheit-Limson EC, Fine J, et al. National Trends and Geographic Variation in Availability of Home Health Care: 2002–2015. *J Am Geriatr Soc*. 2017;65(7):1434-1440. doi:10.1111/jgs.14811

7. HUD USPS ZIP Code Crosswalk Files | HUD USER. Accessed October 31, 2024. https://www.huduser.gov/portal/datasets/usps_crosswalk.html

8. USDA Economic Research Service. Rural-Urban Continuum Codes - Documentation. January 7, 2025. Accessed June 17, 2025. https://www.ers.usda.gov/data-products/rural-urban-continuum-codes/documentation

9. U.S. Department of Justice, the Federal Trade Commission. *Merger Guidelines*.; 2023. Accessed November 14, 2024. https://www.ftc.gov/system/files/ftc_gov/pdf/2023_merger_guidelines_final_12.18.2023.pdf

© 2025 Li K et al. *JAMA Network Open*.
