# Supplementary material for: Corporate Ownership, Health System Affiliation, and Market Concentration of Home Health Agencies
Source: JAMA Netw Open. 2025 Aug 21;8(8):e2528258. doi: 10.1001/jamanetworkopen.2025.28258 (PMC12371508; doi:10.1001/jamanetworkopen.2025.28258)
Supplement: Supplement 2. — Data Sharing Statement [file jamanetwopen-e2528258-s002.pdf]

Data Sharing Statement

Li. Corporate Ownership, Health System Affiliation, and Market Concentration of Home Health Agencies. *JAMA Netw Open*. Published August 21, 2025.
doi:10.1001/jamanetworkopen.2025.28258

Data
Data available: No

Additional Information
Explanation for why data not available: All data used in this study are publicly available.
